# Application of A Convolutional Neural Network in The Diagnosis of Gastric Mesenchymal Tumors on Endoscopic Ultrasonography Images

**DOI:** 10.3390/jcm9103162

**Published:** 2020-09-29

**Authors:** Yoon Ho Kim, Gwang Ha Kim, Kwang Baek Kim, Moon Won Lee, Bong Eun Lee, Dong Hoon Baek, Do Hoon Kim, Jun Chul Park

**Affiliations:** 1 Department of Internal Medicine, Pusan National University School of Medicine and Biomedical Research Institute, Pusan National University Hospital, Busan 49241, Korea; kyho185@naver.com (Y.H.K.); neofaceoff@hanmail.net (M.W.L.); bongsul@hanmail.net (B.E.L.); dhbeak77@gmail.com (D.H.B.); 2 Division of Computer Software Engineering, Silla University, Busan 46958, Korea; gbkim@silla.ac.kr; 3 Department of Gastroenterology, University of Ulsan College of Medicine, Asan Medical Center, Seoul 05505, Korea; kdh0358@hanmail.net; 4 Department of Internal Medicine and Institute of Gastroenterology, Yonsei University College of Medicine, Seoul 03722, Korea; junchul75@yuhs.ac

**Keywords:** stomach, endoscopic ultrasonography, gastrointestinal stromal tumor, mesenchymal tumor, artificial intelligence

## Abstract

Background and Aims: Endoscopic ultrasonography (EUS) is a useful diagnostic modality for evaluating gastric mesenchymal tumors; however, differentiating gastrointestinal stromal tumors (GISTs) from benign mesenchymal tumors such as leiomyomas and schwannomas remains challenging. For this reason, we developed a convolutional neural network computer-aided diagnosis (CNN-CAD) system that can analyze gastric mesenchymal tumors on EUS images. Methods: A total of 905 EUS images of gastric mesenchymal tumors (pathologically confirmed GIST, leiomyoma, and schwannoma) were used as a training dataset. Validation was performed using 212 EUS images of gastric mesenchymal tumors. This test dataset was interpreted by three experienced and three junior endoscopists. Results: The sensitivity, specificity, and accuracy of the CNN-CAD system for differentiating GISTs from non-GIST tumors were 83.0%, 75.5%, and 79.2%, respectively. Its diagnostic specificity and accuracy were significantly higher than those of two experienced and one junior endoscopists. In the further sequential analysis to differentiate leiomyoma from schwannoma in non-GIST tumors, the final diagnostic accuracy of the CNN-CAD system was 75.5%, which was significantly higher than that of two experienced and one junior endoscopists. Conclusions: Our CNN-CAD system showed high accuracy in diagnosing gastric mesenchymal tumors on EUS images. It may complement the current clinical practices in the EUS diagnosis of gastric mesenchymal tumors.

## 1. Introduction

Mesenchymal tumors of the stomach are mostly incidentally observed during routine esophagogastroduodenoscopy examinations, especially as part of the national gastric cancer screening programs in Korea; they appear as hard, elevated subepithelial lesions [1]. The exact incidence of gastric mesenchymal tumors is unknown, but the prevalence of subepithelial tumors detected during routine esophagogastroduodenoscopy was 1.7% (194 of 11,712) in Korea [2]. Histopathologically, most mesenchymal tumors are either entirely or partially made up of spindle cells and demonstrate differentiation of the smooth muscles or nerve sheaths. Most gastric mesenchymal tumors are gastrointestinal stromal tumors (GISTs), which originate from the interstitial cells of Cajal [3,4,5]. GISTs have a metastatic risk, especially to the liver and peritoneum, even after surgical resection for localized disease [6,7]. Therefore, they have considerable malignant potential, and resection is recommended, even when they present as small gastric subepithelial lesions but with possible high-risk endosonographic features such as irregular border, cystic spaces, ulceration, echogenic foci, and heterogeneity [7,8,9,10].

In clinical practice, it is important to differentiate GISTs from benign mesenchymal tumors such as leiomyomas or schwannomas, to manage them appropriately. Endoscopic ultrasonography (EUS) is currently the most useful diagnostic modality for evaluating gastric mesenchymal tumors because it enables clinicians to assess the size, gastric wall layer of origin, margins, echogenicity, and detailed morphology of the lesions [11,12,13]. However, the results of previous studies about differentiating GISTs from benign mesenchymal tumors using EUS have not been consistent [13,14]. There are inevitable limitations in analyzing the characteristic EUS findings of such mesenchymal tumors as a consequence of the subjective interpretation of EUS images by the endoscopists, which finds its expression in poor interobserver agreement [15,16]. Computer-aided diagnosis is expected to help endoscopists overcome these limitations and improve the diagnosis of mesenchymal tumors. In previous studies we showed that digital image analysis using brightness values of tumors in EUS images was useful in diagnosing gastric mesenchymal tumors [1,17].

Recently, image interpretation using artificial intelligence (AI) with machine or deep learning has improved dramatically, and is progressively applied to diverse gastrointestinal endoscopy fields [18,19]. Machine learning refers to the scientific study of algorithms and statistical models with which a computer can learn on its own using multiple datasets, without being provided explicit instructions; deep learning is a subfield of machine learning algorithms and uses layers of nonlinear processing for feature extraction and transformation from unstructured or unlabeled data [19]. The convolutional neural network (CNN), a deep learning algorithm, is a neural network currently considered to possess the best performing image recognition algorithm [20,21]. Accordingly, deep learning via a CNN using comprehensive image data holds a high potential for clinical application in analyzing medical images. For this reason, we decided to investigate the values of CNN in the differentiation of GISTs from benign mesenchymal tumors on EUS images. We assessed the performance of a novel sequential system based on CNN that we had developed for analyzing gastric mesenchymal tumors observed during EUS.

## 2. Methods

### 2.1. Training and Test Image Datasets

We obtained EUS images from three hospitals (Pusan National University Hospital, Busan, Korea; Asan Medical Center, Seoul, Korea; and Severance Hospital, Seoul, Korea) that had been recorded between January 2007 and June 2018 for retrospective analysis. EUS images were captured using radial-scanning echoendoscopes (GF-UM2000 and GF-UE260P, Olympus Medical Systems, Corp., Tokyo, Japan) at 7.5 MHz and ultrasound processors (EU-ME1, Olympus Medical Systems; and ALOKA Prosound Alpha-10 processor, ALOKA Company, Ltd., Tokyo, Japan).

The inclusion criteria for EUS images were the display of gastric GISTs, leiomyomas, and schwannomas, which had been histopathologically confirmed by surgical or endoscopic resection and/or EUS-guided fine-needle biopsy (EUS-FNB). The exclusion criterion was poor quality resulting from blur or lack of focus. After applying these criteria, we used 587 images of 179 gastric mesenchymal tumors as the training image dataset (428 images of 125 GISTs, 91 images of 33 leiomyomas, and 68 images of 21 schwannomas) (Table 1). At least one entire tumor lesion was visible on all images, and multiple images were assembled for the same lesion to include different angles and distances.

We then collated an independent test dataset of 212 EUS images of 69 gastric mesenchymal tumors that had been recorded at the same institutions from July 2018 to June 2019 (106 images of 32 GISTs, 60 images of 23 leiomyomas, and 46 images of 14 schwannomas). All EUS findings had been histopathologically confirmed as either GISTs, leiomyomas, or schwannomas by surgical or endoscopic resection or EUS-FNB.

The protocol of this study was reviewed and approved by the Pusan National University Hospital Institutional Review Board (IRB number, H-2002-026-088)

### 2.2. Histopathology

The tumors were immunohistochemically classified into leiomyomas, schwannomas, or GISTs, as previously described [5,22]. GIST was defined as a c-kit (CD117), CD34, or DOG-1-positive tumor. Leiomyoma was defined as a desmin-positive and c-kit-negative tumor, while schwannoma was defined as an S-100-positive and c-kit-negative tumor. The mesenchymal tumors were then classified into two groups according to their malignant potential: the GIST and non-GIST tumor (leiomyoma and schwannoma) groups

### 2.3. Mesenchymal Tumor Classification Algorithm

#### 2.3.1. Tumor Dataset Generation

**Figure 1:**
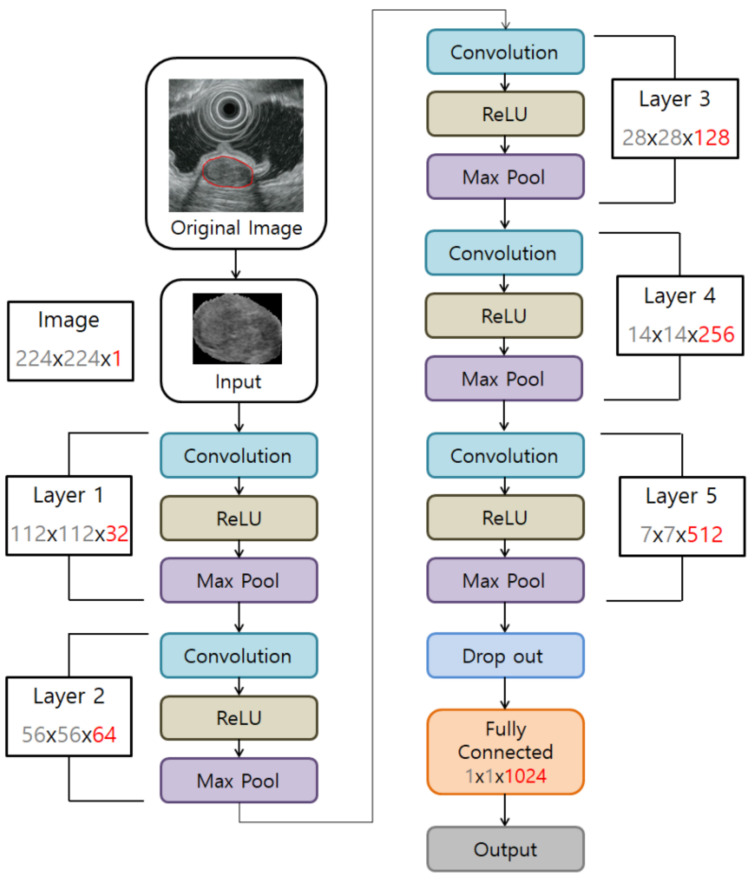
Convolutional neural network computer-aided detection system architecture.

Tumor regions were selected from the EUS images to generate image datasets for deep learning models. All images of mesenchymal tumor lesions were marked manually by an experienced endoscopist (GH Kim), who was blinded to the final diagnosis. All the selected areas with carefully drawn outer margins of tumors (using a mouse) were used as the dataset (Figure 1).

#### 2.3.2. Dataset Configuration

The initial training dataset of 587 EUS images described above contained more images of GISTs than of leiomyomas and schwannomas. Therefore, the neural network learning was focused on GISTs, whereas leiomyomas and schwannomas might not have been appropriately learned. To compensate for this, we used data augmentation to balance the dataset [23]. It is reported that data augmentation is effective in improving the performance of image classification, and has been applied in various image recognition studies in humans [24,25]. Hence, we used data augmentation exclusively on the training dataset but not on the test dataset. Accordingly, the training dataset images of leiomyomas and schwannomas were inverted up and down and left and right to increase the amount of image data by three times. Consequently, the final training dataset used in our model to differentiate GISTs from non-GIST tumors comprised 905 EUS images (428 images of GISTs, 273 images of leiomyomas, and 204 images of schwannomas).

We also tried to differentiate leiomyomas from schwannomas within the non-GIST tumors, but the number of images in the training dataset was small (91 images of leiomyomas and 68 images of schwannomas). Therefore, the training dataset images of leiomyomas and schwannomas were also inverted up and down and left and right (schwannoma only). This yielded a final training dataset used in the model to differentiate leiomyomas from schwannomas of 545 EUS images (273 images of leiomyomas and 272 images of schwannomas).

#### 2.3.3. CNN Algorithm Construction to Develop a Tumor Classification Model

Our CNN algorithm consisted of convolution, subsampling, and full connection phases. Its overall structure is shown in Figure 1. Any image coming in as an input passed through several filters in the convolution stage to create a feature map for each filter. Once the feature map was created, it went through the subsampling step. The feature map reduced by subsampling was input to the next convolution layer to be formed again by passing through the filters, and the same operation was repeated for each layer. When convolution and pooling were completed, the output value was calculated based on the final features to arrive at the classification result. The CNN constructed in this study consisted of five layers, and the input image was a black and white image of 224 × 224 pixels. The output layer was firstly classified into two groups—GISTs and non-GIST tumors. In the non-GIST tumor group, the output layer was then further subclassified into schwannoma or leiomyoma.

### 2.4. Outcome Measures

After constructing the CNN computer-aided diagnosis (CNN-CAD) system using the training dataset, we evaluated the performance with the test dataset. The classification provided by the CNN-CAD system was compared with the histopathology results. Receiver operating characteristic (ROC) curves were constructed by varying the operating threshold. Sensitivity, specificity, positive predictive value (PPV), negative predictive value (NPV), and accuracy with a 95% confidence interval (CI) were calculated using standard definitions.

We then compared the classification performance of the CNN-CAD system with that of the endoscopists. To achieve this, six endoscopists of varying experience interpreted the EUS images of the mesenchymal tumors in the test dataset. Among them, three endoscopists were classified as experienced, having performed more than 500 EUS examinations of gastric subepithelial tumors, whereas the others were classified as junior endoscopists who had performed less than 200 of these EUS examinations. The endoscopists received the original EUS images without any comments and provided their own classification of every EUS image without knowledge of the CNN-CAD system classification results. The classification by the endoscopists was also compared with the final histopathology results.

### 2.5. Statistical Analysis

We used the two-sided McNemar tests to compare the sensitivity, specificity, PPV, NPV, and accuracy between endoscopists and the CNN-CAD system. One of the authors (DH Baek), who was not involved in the development of the CNN-CAD system, performed the comparative statistical analysis of the classification performance between the CNN-CAD system and the endoscopists. The statistical analyses were conducted using the language R (http://cran.r-project.org) version 3.5.0 and additional packages (DescTools, qwraps2). A *p* value < 0.05 was considered statistically significant.

## 3. Results

### 3.1. Performance of the CNN-CAD System in the Training Dataset

In the training dataset of 905 images of gastric mesenchymal tumors, the CNN-CAD system diagnosed 443 tumors as GISTs and 462 as non-GIST tumors. It correctly identified 338 of the 428 GISTs and 372 of the 477 non-GIST tumors. The sensitivity, specificity, PPV, NPV, and accuracy of the CNN-CAD system to differentiate GISTs from non-GIST tumors were 79.0% (95% CI: 75.9–81.8%), 78.0% (95% CI: 75.2–80.5%), 76.3% (95% CI: 73.3–79.0%), 80.5% (95% CI: 77.7–83.1%), and 78.5% (95% CI: 75.5–81.1%), respectively.

Among the 545 images of non-GIST tumors, the CNN-CAD system diagnosed 249 tumors as leiomyomas and 296 as schwannomas. It correctly identified 220 of the 273 leiomyomas and 243 of the 272 schwannomas. The sensitivity, specificity, PPV, NPV, and accuracy of the CNN-CAD system to differentiate leiomyomas from schwannomas were 89.3% (95% CI: 86.0–92.1%), 80.6% (95% CI: 77.2–83.4%), 82.1% (95% CI: 79.0–84.6%), 88.4% (95% CI: 84.7–91.4%), and 85.0% (95% CI: 81.6–87.7%), respectively.

### 3.2. Performance of the CNN-CAD System in the Test Dataset

**Figure 2:**
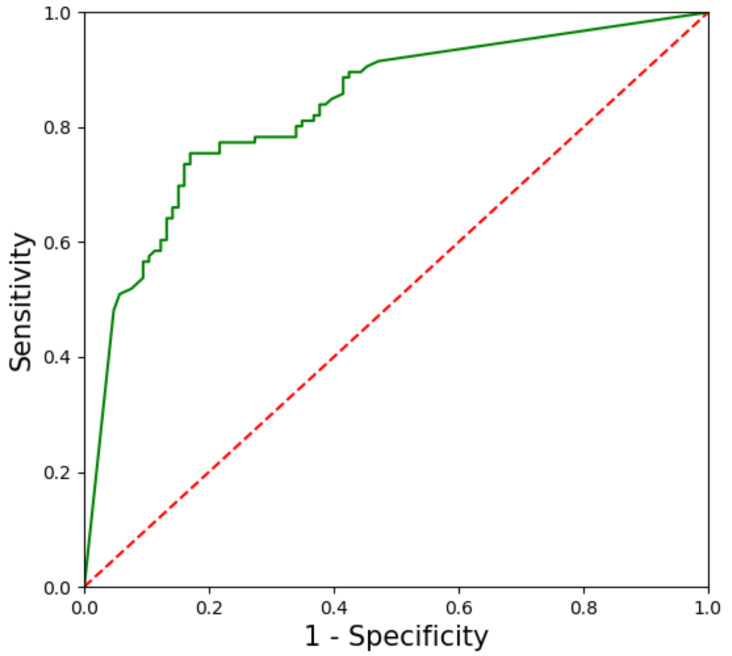
Receiver operating characteristic curve for the test dataset. The area under the curve is 83.4%.

In the test dataset, the CNN-CAD system required 8.2 s to analyze the 212 images (at a speed of 26 images per second). The CNN-CAD system diagnosed 114 tumors as GISTs and 98 as non-GIST tumors. It correctly identified 88 of the 106 GISTs and 80 of the 106 non-GIST tumors. To assess the robustness of our CNN-CAD system, a ROC curve was plotted for each test image based on the probability of a tumor being classified as either a GIST or non-GIST tumor (Figure 2). The area under the curve was 0.834 (95% CI, 0.779–0.889). The sensitivity, specificity, PPV, NPV, and accuracy of the CNN-CAD system to differentiate GISTs from non-GIST tumors were 83.0% (95% CI: 76.6–88.2%), 75.5% (95% CI: 69.1–80.7%), 77.2% (95% CI: 71.2–82.0%), 81.6% (95% CI: 74.7–87.3%), and 79.2% (95% CI: 72.8–84.5%), respectively (Table 2). There was no significant difference in the sensitivity of diagnosis between the CNN-CAD system and endoscopists. However, the diagnostic specificity and accuracy of the CNN-CAD system were significantly higher than those of two experienced and one junior endoscopists (all *p* < 0.05).

**Figure 3:**
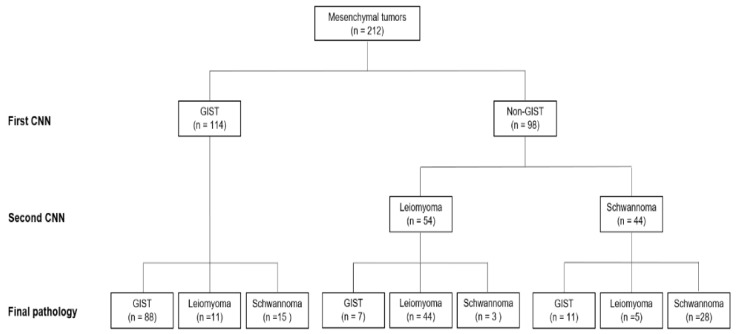
Flowchart of sequential convolutional neural network computer-aided detection system in the test dataset.

We then performed a further sequential analysis of the 98 tumors classified as non-GIST tumors with the CNN-CAD system to differentiate leiomyomas from schwannomas; 54 tumors were diagnosed as leiomyomas and 44 as schwannomas (Figure 3). The final accuracy of our sequential CNN-CAD system for gastric mesenchymal tumors was 75.5% (95% CI: 69.3–80.8%) (Table 3). It correctly diagnosed 88 (83.0%) of the 106 GISTs, 44 (73.2%) of the 60 leiomyomas, and 28 (60.9%) of the 46 schwannomas. The diagnostic accuracy of the CNN-CAD system was significantly higher than that of two experienced and one junior endoscopists (all *p* < 0.05).

## 4. Discussion

In this study, we present the results of a newly developed AI-based diagnostic system in differentiating GISTs from benign mesenchymal tumors on EUS images using a CNN that simulates the human brain. Our CNN-CAD system differentiated GISTs from non-GIST tumors with a sensitivity of 83.0% and a specificity of 75.5% in the independent test dataset. Furthermore, the sequential CNN-CAD system could predict the histopathology of gastric mesenchymal tumors with an accuracy rate of 75.5%. To the best of our knowledge, this is the first study to report on the use of a CNN-CAD system to differentiate gastric mesenchymal tumors on EUS images.

In our previous studies, we reported on the characteristic EUS features of GISTs, leiomyomas, and schwannomas [13,26]. Heterogeneity, hyperechogenic spots, a peripheral halo, and higher echogenicity when compared with those in the normal proper muscle layer were the useful criteria in differentiating GISTs from other mesenchymal tumors. However, the interpretation of EUS findings is subjective and depends on the endoscopists.

Our CNN-CAD system analyzed gastric mesenchymal tumors sequentially based on a CNN; the first step was to classify the gastric mesenchymal tumors into GISTs and non-GIST tumors and then to sub-classify non-GIST tumors into leiomyomas and schwannomas. In the first step, our results showed that the specificity and accuracy of the CNN-CAD system were better than those of two experienced and one junior endoscopists, while there was no significant difference in the sensitivity between the CNN-CAD system and all of the endoscopists. In the second step, the testing results showed that the accuracy of the CNN-CAD system was also better than that of two experienced and one junior endoscopists. Hence, the CNN-CAD system performed similarly or better in detecting the specific EUS features of mesenchymal tumors than the endoscopists, and has the potential to provide diagnostic assistance to endoscopists in the future.

The diagnostic capability of two of the experienced endoscopists was not as good as that of the junior endoscopists in our study. Paradoxically, an extensive period of training and practice to become skilled and proficient in the clinical application of EUS does not seem to facilitate the diagnosis of gastric mesenchymal tumors. Therefore, more invasive procedures for tissue acquisition, such as EUS-FNB and submucosal dissection-assisted deep biopsy, are sometimes recommended in clinical settings to evaluate the malignant potential of gastric mesenchymal tumors [1]. This is another reason for the introduction of a CNN-CAD system: a further CNN-CAD may help to reduce diagnostic errors made by endoscopists and lead to the development of optimal treatment plans.

**Figure 4:**
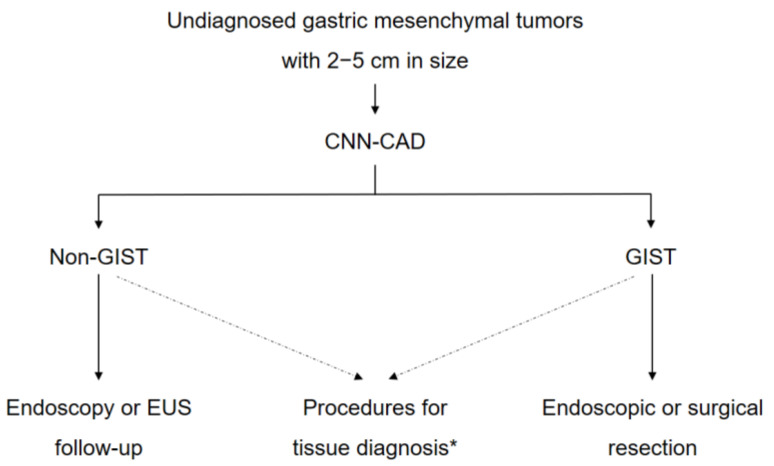
A suggested strategy based on the convolutional neural network computer-aided diagnosis system for gastric mesenchymal tumors of 2–5 cm in size. CNN-CAD, convolutional neural network computer-aided detection; EUS, endoscopic ultrasonography; GIST, gastrointestinal mesenchymal tumor. *EUS-guided or submucosal dissection-assisted biopsy.

The problem that remains is the application of this CNN-CAD system in making real-world clinical decisions in gastric mesenchymal tumors. Recent guidelines recommend follow-up using endoscopy or EUS once or twice a year for gastric mesenchymal tumors that are <2 cm in size without high-risk EUS features until they are symptomatic or grow in size [10,27,28]. In contrast, surgical resection is recommended for gastric mesenchymal tumors >5 cm in size. However, for gastric mesenchymal tumors of 2–5 cm in size, invasive procedures such as EUS-guided or endoscopic submucosal dissection-assisted biopsy are generally recommended to obtain histopathological confirmation and differentiate GISTs from non-GIST tumors. In real-world practice, the management of gastric mesenchymal tumors of 2–5 cm in size can be problematic to both endoscopists and patients. We believe that the CNN-CAD system could be useful in such cases in choosing the next diagnostic or treatment modality (Figure 4).

This study has several limitations. First, the training dataset and test dataset images were relatively small in number, and all EUS images were still images retrospectively obtained from only three centers. The results of retrospective studies tend to be better than what can be achieved in real-life settings because of selection bias. The types of echoendoscopes and ultrasound processors are highly variable across different facilities. Furthermore, we used only high-quality EUS images for the training and test datasets. If the images are blurred or show a lack of focus, the CNN-CAD system is likely to make mistakes—although the same has been observed with endoscopists [29]. Therefore, we think that the ability of the CNN-CAD system to evaluate poor-quality EUS images should be investigated after its performance has been confirmed in large numbers of high-quality images. Lastly, we analyzed the diagnostic performance of the CNN-CAD system in each EUS image, not in each lesion. In addition, the proportion of GISTs in the test dataset was lower than that in the training dataset (69.8% vs. 46.4%). These factors would have influenced the diagnostic performance of the CNN-CAD system and endoscopists.

Despite these limitations, our results are valuable in that the CNN-CAD system can differentiate GISTs from non-GIST tumors within a short amount of time and with high sensitivity and specificity. We currently plan to conduct a large-scale, multi-center prospective study to validate the diagnostic ability of the CNN-CAD system to predict the histopathology of gastric mesenchymal tumors.

In conclusion, we developed a CNN-CAD system for diagnosing gastric mesenchymal tumors with high accuracy and specificity. It distinguished GISTs from non-GIST tumors, which may complement current clinical practice in the EUS diagnosis of gastric mesenchymal tumors.

## Figures and Tables

**Table 1:** Clinicopathological characteristics of patients with gastric mesenchymal tumors in the training and test datasets.

Characteristics	Training Dataset (*n* = 179)	Test Dataset (*n* = 69)
Age, Years (mean ± SD)	57.7 ± 11.9	56.9 ± 13.7
Sex		
Male	80 (44.7)	31 (44.9)
Female	99 (55.3)	38 (55.1)
Tumor Location		
Upper	105 (58.7)	43 (62.3)
Middle	57 (31.8)	21 (30.4)
Lower	17 (9.5)	5 (7.2)
Tumor Size, cm (mean ± SD)	3.6 ± 2.1	3.2 ± 1.6
Final Histopathology		
Leiomyoma	33 (18.4)	23 (33.3)
Schwannoma	21 (11.7)	14 (20.3)
Gastrointestinal Stromal Tumor	125 (69.8)	32 (46.4)
Very Low Risk	27	7
Low Risk	50	14
Intermediate Risk	29	7
High Risk	19	4

Values are *n* (%).

**Table 2:** Diagnostic performance of a convolutional neural network computer-aided detection system compared to that of endoscopists in differentiating gastrointestinal stromal tumors (GISTs) from non-GIST tumors.

	CNN-CAD System	Endoscopists					
		Experienced 1	Experienced 2	Experienced 3	Junior 1	Junior 2	Junior 3
Sensitivity, %	83.0 (77.4–87.5)	83.0 (77.4–87.5)	74.5 (68.3–79.9)	71.7 (65.3–77.3)	84.0 (78.4–88.3)	73.6 (67.3–79.1)	84.9 (79.5–89.1)
Specificity, %	75.5 (69.3–80.8)	68.9 (62.3–74.7)	61.3 * (54.6–67.6)	56.6 * (49.9–63.1)	63.2 (56.5–69.4)	77.4 (71.3–82.5)	53.8 * (47.1–60.4)
Positive predictive value, %	77.2 (71.1–82.3)	72.7 (66.4–78.3)	65.8 (59.2–71.9)	62.3 * (55.6–68.5)	69.5 (63.0–75.3)	76.5 (70.3–81.7)	64.7 * (58.1–70.9)
Negative predictive value, %	81.6 (75.9–86.3)	80.2 (74.3–85.0)	70.7 (64.2–76.4)	66.7 * (60.1–72.7)	79.8 (73.8–84.6)	74.5 (68.3–79.9)	78.1 (72.0–83.1)
Accuracy, %	79.2 (73.3–84.2)	75.9 (69.8–81.2)	67.9 * (61.4–73.8)	64.2 * (57.5–70.3)	73.6 (67.3–79.1)	75.5 (69.3–80.8)	69.3 * (62.8–75.2)

Values in parenthesis are 95% confidence intervals. CNN-CAD, convolutional neural network computer-aided detection. * Significant differences compared with the CNN-CAD system (*p* < 0.05).

**Table 3:** Overall diagnostic accuracy of gastric mesenchymal tumors by a convolutional neural network computer-aided detection system compared to that of endoscopists.

	CNN-CAD System	Endoscopists					
		Experienced 1	Experienced 2	Experienced 3	Junior 1	Junior 2	Junior 3
Accuracy, %	75.5 (69.3–80.8)	72.6 (66.3–78.2)	61.8 * (55.1–68.1)	59.0 * (52.2–65.4)	67.0 (60.4–73.0)	68.4 (61.9–74.3)	66.0 * (59.4–72.1)

Values in parenthesis are 95% confidence intervals. CNN-CAD, convolutional neural network computer-aided detection. * Significant differences compared with CNN-CAD system (*p* < 0.05).
